# Benefits of the Mediterranean diet beyond the Mediterranean Sea and beyond food patterns

**DOI:** 10.1186/s12916-016-0714-3

**Published:** 2016-10-14

**Authors:** Miguel A. Martínez-González

**Affiliations:** 1 Department of Preventive Medicine and Public Health, University of Navarra, IdiSNA, Pamplona, Spain; 2 Department of Nutrition, Harvard T.H. Chan School of Public Health, Boston, MA USA; 3 CIBER Fisiopatología de la Obesidad y la Nutrición (CIBEROBN), Instituto de Salud Carlos III, Madrid, Spain

**Keywords:** Mediterranean diet, Cardiovascular disease, Primary prevention, Obesity, Clinical trial

## Abstract

Abundant and growing evidence has accrued to demonstrate that the traditional Mediterranean diet is likely to be the ideal dietary pattern for the prevention of cardiovascular disease. A landmark randomized trial (PREDIMED) together with many well-conducted long-term observational prospective cohort studies support this causal effect.

A new, large British cohort study by Tong et al. assessing the association between adherence to the Mediterranean diet and cardiovascular disease was recently published in *BMC Medicine*. Using a superb methodology, they followed-up 23,902 participants for 12.2 years on average and observed several thousand incident cases.

The results of this cohort study showed a significant beneficial effect of the Mediterranean diet on cardiovascular events. These findings support the transferability of this dietary pattern beyond the shores of the Mediterranean Sea. The authors provided measures of population impact in cardiovascular prevention and estimated that 19,375 cases of cardiovascular death would be prevented each year in the UK by promoting the Mediterranean Diet.

Please see related article: http://bmcmedicine.biomedcentral.com/articles/10.1186/s12916-016-0677-4.

## Background

The huge toll of death and disability caused each year by cardiovascular disease (CVD) continues to represent a great humiliation for public health. More people die annually from CVDs than from any other cause, and global projections are somber with a projected increase from 17 million to more than 24 million yearly CVD deaths in 2030 [1, 2]. Not a minor part of this humiliation comes from the knowledge that CVD can largely be prevented by timely population changes in metabolic risk factors including obesity, hypertension, dyslipidemia, and diabetes. This unambiguously indicates the need to address unhealthy lifestyles and replace unhealthy food patterns with their healthiest alternatives [3].

In this context, strong evidence supports the traditional Mediterranean diet (MedDiet) as the optimal choice for preventing CVD [3, 4]. Two decades ago, the Lyon diet-heart study, a randomized controlled clinical trial, was unique in showing that a dietary intervention brought about a dramatic reduction in cardiovascular events among survivors of a previous myocardial infarction [5]. The dietary intervention was based on the MedDiet. Subsequently, many prospective cohort studies have consistently confirmed the cardiovascular benefits of the traditional MedDiet. These cohort studies (pooled in different meta-analyses) constitute an impressive accrual of high-quality epidemiological evidence, currently not available for any other dietary pattern [6–11] and meet the classical criteria for causality in epidemiology [12]. However, for a long time, the only available evidence on cardiovascular prevention by the MedDiet was exclusively based on observational designs (with the single exception of the Lyon study). Observational studies can be affected by some biases, particularly measurement errors and residual confounding. But, this potential objection was only tenable until 2013, when the final results of the PREDIMED (“Prevencion con Dieta Mediterranea”) primary prevention trial were published [13]. This landmark trial included 7447 participants and showed a 30 % relative reduction in the risk of hard CVD events during 4.8 years of follow-up. Most hesitations about and criticisms of previous observational designs were removed by the almost perfect consistency between well-conducted observational cohort studies and trials. This consistency is of paramount importance for supporting the validity of well-conducted observational cohort studies in nutritional epidemiology as well as for evidence-based health promotion [8, 12, 14].

A review summarizing only the cohort studies published during 2015 and the first months of 2016 identified 19 new prospective studies on the traditional MedDiet [15]. Substantial benefits were observed with regards to myocardial infarction, stroke, heart failure, and total mortality.

The pending challenge was to test if the proven benefits of the MedDiet were restricted to populations living in Mediterranean areas or whether they were fully transferable far beyond the shores of the Mediterranean Sea. An excellent positive answer to this question is found in the new results from the EPIC-Norfolk cohort study [16]. In a UK general population setting, Tong et al. evaluated 23,902 participants followed-up for 12.2 years on average and observed several thousand new cases of CVD and 1714 CVD deaths. MedDiet adherence (repeatedly evaluated during follow-up) was inversely associated with hard clinical cardiovascular events (a composite end-point including ischemic heart disease [IHD], ischemic stroke, hemorrhagic stroke, heart failure, peripheral vascular disease, and other CVD events) [16]. The design, conduction, epidemiological/statistical analyses, and interpretation of findings in this study are superb. The authors are to be commended for their excellent work. Interestingly, they included absolute measures of risk reduction, potential impact (population attributable fractions [PAF]), and number needed to treat, under the assumption of a truly causal protective effect of the MedDiet on CVD. Approximately 8.5 % of incident IHD or stroke events in the UK were attributable to low adherence to the MedDiet (equivalent to 10.2 IHD or stroke cases preventable per 1000 population). They estimated that 19,375 cases of CVD death would be prevented each year by promoting the MedDiet in the UK.

Further strengths of the study by Tong et al. [16] include its long-term follow-up, optimal ascertainment of outcomes, careful adjustment for a wide array of potential confounders, considerations to potential competing risks, and, in particular, the diverse sensitivity analyses using different definitions of the MedDiet. It is reassuring that benefits were similar upon alternative categorizations of food groups in four scores of the MedDiet. However, the profile of a traditional MedDiet may be different in the UK than in Southern European countries and further questions about transferability still remain. This may explain why some magnitudes of effect were inferior to those from the PREDIMED trial [9].

It is a fact that culinary practices, average intakes (and consequently the cut-off points used for computing scores), serving sizes, and combinations of foods are likely to be different in studies conducted in Mediterranean settings than in those conducted beyond the shores of the Mediterranean Sea. Despite this, Tong et al. have presented an excellent study that has obtained salient results with practical consequences for nutrition policy after using food frequency questionnaires (FFQs) habitually applied in sound studies of nutritional epidemiology. Well-validated FFQs represent a valid and realistic tool for nutritional assessment in large longitudinal studies and randomized trials in nutrition [14].

## Conclusions and future directions

Practical consequences of the study by Tong et al. for nutrition policies are that healthy natural fats typical of the MedDiet (extra-virgin olive oil, tree nuts, and fatty fish) can now be more confidently promoted in the UK. We should also allay fears regarding a moderate and usual consumption of red wine spread out over the week, following the traditional Mediterranean alcohol-drinking pattern [17]. Also, there are strong reasons to promote an abundance of plant foods, especially fresh fruits as the usual dessert. Conversely, there is a need to substantially reduce the consumption of meat, especially red and processed meats, ice creams, fizzy soft drinks, sugary desserts, sweet biscuits, other commercial bakery, cream pies and cakes [16].

The first future direction in research is that a large randomized trial with MedDiet and hard clinical events is needed beyond the Mediterranean Sea. The US National Institutes of Health are planning to soon launch such a trial in the US [18]. This initiative is very much welcomed and there is preliminary evidence in favor of the transferability of the MedDiet (including extra virgin olive oil and tree nuts) to the US [19].

Second, there is a need to go not only beyond the Mediterranean Sea (as the EPIC-Norfolk study did) but also beyond the MedDiet and beyond food patterns. After the successful results of interventions with MedDiet, even if weight loss, caloric restriction, or physical activity were not included [9], it is very likely that an energy-restricted MedDiet with physical activity and weight loss might be an optimal intervention. A large lifestyle multifactorial trial going beyond food patterns would be of high interest. There is, in fact, an urgent need for such a trial because most doctors are setting goals for weight loss and exercise for their overweight patients, but there is no large long-term randomized trial showing a reduction in cardiovascular clinical events with these interventions.

Fortunately, a large trial focused on MedDiet, weight loss, and physical activity (PREDIMED-PLUS) is ongoing, and it has already randomized 6000 participants [20]. The results of the EPIC-Norfolk study and the expected results of PREDIMED-PLUS may represent powerful evidence-based answers to what is currently the most important question for public health: how to effectively tackle chronic diseases. These answers are likely to be strong enough to eventually reverse the deep humiliation for public health that the burden of CVD represents.
